# Diagnostic sensitivity of fine-needle aspiration cytology in thyroid cancer

**DOI:** 10.1038/s41598-024-75677-7

**Published:** 2024-10-16

**Authors:** Patrik Lind, Anton Jacobson, Erik Nordenström, Lars Johansson, Göran Wallin, Kosmas Daskalakis

**Affiliations:** 1 Anesthesiology Department, Skellefteå Hospital, Skellefteå, Sweden; 2 Department of Surgery, Faculty of Medicine and Health, Örebro University, Örebro, 70185 Sweden; 3 Department of Surgery, Skåne University Hospital, Lund, Sweden; 4 Department of Clinical Sciences, Lund University, Lund, Sweden; 5 Department of Public Health and Clinical Medicine, Skellefteå Research Unit, Umeå University, Umeå, SE-901 81 Sweden; 6 Second Department of Surgery, Korgialenio-Benakio, Red Cross General Hospital, Athanasaki 11, Athens, 11526 Greece

**Keywords:** Diagnostic sensitivity, Fine-needle aspiration cytology, Thyroid cancer, Cancer, Thyroid diseases, Cancer, Medical research

## Abstract

Introduction Fine-needle aspiration cytology (FNAC) is an effective tool in the diagnostic work-up of patients with thyroid nodules. The aim of our study was to assess the diagnostic sensitivity of FNAC in thyroid cancer (TC) in Sweden by correlating the findings of preoperative FNAC with those obtained through final histology of the surgical specimen. Methods A Swedish nationwide cohort of patients having surgery for TC (n = 2519) from the Scandinavian Quality Register for Thyroid, Parathyroid and Adrenal surgery between 2004 and 2013 was obtained. Data was validated through scrutinizing patient FNAC and histology reports. Results Among the 2519 cases operated with a final diagnosis of TC, the diagnosis was substantiated and validated through the histology report in 2332 cases (92.6%), included in the present study. Among these, 1679 patients (72%) were female and the median age at TC diagnosis was 52.3 years (range 18-94.6). In 353 cases (15.1%) FNAC was not performed at all; whereas in the remaining 1965 cases, the diagnostic sensitivity of FNAC was 81.6%. In lesions > 1 cm, FNAC diagnostic sensitivity reached 86.5%, whereas in lesions < 1 cm, FNAC yielded a sensitivity of 61.5%. Approximately 85% of FNACs (n = 1981/2332) were performed using ultra-sonographic (US) guidance. In TC lesions > 1 cm, the diagnostic sensitivity of US-guided FNAC (n = 1504) was 86.9% as compared to 76.9% in clinically applied FNAC without US utilization (n = 118). Conclusions FNAC is performed in most patients operated for TC in Sweden (85%) and retains its value as a tool in TC diagnostic work-up with an overall sensitivity of 82%, reaching 87% in lesions > 1 cm, that harbor clinically relevant TC.

**Supplementary Information:**

The online version contains supplementary material available at 10.1038/s41598-024-75677-7.

## Introduction

Thyroid cancer (TC)is the most common endocrine malignancy and its incidence is rising at a rate 5–6% per year in the US, as well as internationally in analysis of population-level cancer registries world-wide^1–3^. Although the prognosis of well-differentiated TC (WDTC) cases, which constitute the majority of TCs (94–98%), is generally favorable, disease-specific mortality has been increasing at an average rate of 0.9% per year^1,3^. Despite that the observed increase in TC incidence appears to be coincident with early detection due to the widespread use of neck ultrasonography and fine-needle aspiration cytology (FNAC), this may not be the only reason for rising incidence rates, as this trend is observed across all different stages, among all ages, genders and ethnic groups^4^.

Malignant follicular cell–derived neoplasms (FDTC) include papillary (PTC), follicular (FTC), and oncocytic (OTC) cell cancers that arise from follicular epithelial cells. High-grade FDTC now includes both the traditional poorly differentiated carcinoma, as well as high-grade differentiated thyroid carcinomas since both are characterized by increased mitotic activity and tumor necrosis without anaplastic histology and clinically behave in a similar manner^5^. Anaplastic thyroid carcinoma (ATC) remains the most undifferentiated form with a dismal prognosis. Medullary thyroid carcinoma (MTC) arises from parafollicular C-cells which produce calcitonin, and accounts for 5% of thyroid cancers, whereas less than 1–3% of TCs are ATC^5–7^.

FNAC is one of the most useful methods for preoperative diagnosis of TC in clinical practice. A combined approach of neck ultrasound and FNAC comprise the main diagnostic workup of thyroid lesions. In recent years, standardization of diagnostic terminology following the Bethesda System for Reporting Thyroid Cytology has been widely adopted to assist clinicians and provide an evidence-based approach in the management of thyroid lesions^8,9^. Importantly, there has been a great controversy in the management of thyroid nodules within certain Bethesda categories, particularly in the indeterminate categories of atypia of undetermined significance/follicular lesion of undetermined significance (AUS/ FLUS), as well as suspicious for follicular neoplasm lesions^10–12^. These indeterminate lesions pose various diagnostic difficulties, as they demonstrate a broad range of histologic outcomes spanning benign to malignant entities with wide variations in malignancy rates^12^.

The aim of the present study was to assess the sensitivity of FNAC in the setting of TC through registry data at a nation-wide level and in respect of the rates of malignancy in cytology reports and final histology. We aimed to assess how often FNAC was applied in the diagnostic work-up of TC and the diagnostic sensitivity of FNAC in respect of final histology of the surgical specimen.

## Materials and methods

The study was approved by the regional ethical committee in Uppsala and the Swedish Ethical Review Authority (2012/055 and 2020–05783). Data were primarily collected from the Scandinavian Quality Register for Thyroid, Parathyroid and Adrenal Surgery (SQRTPA) and subsequently validated through scrutinizing FNAC and histology reports across 37 hospitals in Sweden. All patients registered in SQRTPA have provided informed consent to participate in our registry-based study and the consent has been documented and stored in the patients’ medical records. According to the Ethical Approval decision, waiver of informed consent was obtained for further data validation and analysis of the existing SQRTPA data. All methods were carried out in accordance with relevant national guidelines and institutional regulations.

SQRTPA contains data on patient demographics, preoperative, as well as operative and postoperative data, histopathological information, and follow-up information at 6 weeks and 6 months after surgery; with a 90% coverage for thyroid procedures at a national level. Data on adult patients (> 18 years) with a postoperative histopathological diagnosis of TC operated upon between 1 January 2004 and 31 December 2013 were extracted from the SQRTPA. Subsequently, registry data was validated through scrutinizing FNAC and histology reports to identify registry misclassification/misdiagnoses and finalize inclusion to the study. In addition, a questionnaire for all included patients in this study was sent to the attending surgeons in the SQRTPA participating departments and pertinent data was extracted (Supplement 1). Only patients with available histopathological reports and definite histopathological diagnosis of TC on primary thyroid surgery were included. Patients subjected to completion surgery during the study period without available data though from the primary procedure (histopathology and preoperative cytology) were excluded.

During the study period, we did not have a standardized ultrasonography (US) classification system for TC cases, such as Thyroid Imaging Reporting and Data System (TIRADS) or European Thyroid Imaging Reporting and Data System (EU-TIRADS). Generally, in the Swedish clinical practice between 2004 and 2013, thyroid lesions having at least 1 suspicious US feature (i.e., a non-oval shape, irregular margins, microcalcifications, or marked hypoechogenicity) were considered at high risk of malignancy, and the risk was increasing with the number of suspicious features. FNAC was commonly performed for nodules > 10 mm and suspicious US features, although sub-centimenter lesions were occasionally subjected to FNAC.

Cytological diagnoses were categorized into five groups: non-diagnostic, benign, AUS/FLUS and follicular neoplasm, suspicious for malignancy and malignant FNAC. Since it requires histologic evidence of capsular/vascular invasion to distinguish follicular adenoma from follicular carcinoma; nodules selected for surgery may have other clinical or ultrasonographic features that increase suspicion; and a substantial part of the present cytological data (FNAC reports) was obtained prior to the world-wide Bethesda adoption and its introduction to the Swedish clinical practice, we could not always distinguish AUS/FLUS and follicular neoplasm categories and fully adapt our data to the latest 2023 Bethesda System for Reporting Thyroid Cytopathology^13^.We used the 5th edition of the 2022 WHO Classification of Endocrine and Neuroendocrine Tumors that relate to the thyroid gland to classify TC types^5^.

In respect of surgical indications in this cohort during the study period, all intermediate FNAC categories, i.e. patients with AUS/FLUS and follicular neoplasm results were subjected to surgery, mainly diagnostic lobectomies and were not considered for repeat biopsy and/or additional adjunct testing like molecular testing, which was not available then. In addition, although FNAC in lesions < 1 cm was not generally recommended, positive for malignancy FNACs commonly resulted in referrals for surgery. Notably, during the inclusion process we excluded all cases with unavailable histology report as well as cases of completion thyroidectomies (*n* = 67) with unavailable cytology and/or histology data on the primary operation, e.g. cases primarily operated prior to the inclusion period with incomplete data. Apart from completion procedures, 87 cases reported as TC in SQRTPA, had a benign histology and therefore were considered misclassifications.

For the calculation of FNAC sensitivity, thyroid lesions with indeterminate (includes AUS/FLUS, FN, or SM) or malignant FNAC results that were found to be malignant at the time of surgery were considered true-positive FNAC results, whereas lesions with benign FNAC and malignant final histology, as false-negative FNAC results. The formula: *Sensitivity = true positives / (true positives + false negatives)* was used to compute sensitivity rates in our dataset. However, as nodules with ultimately benign histology were not included in the study cohort, we were not able to compute FNAC diagnostic specificity and accuracy due to the lack of data on true negative and false-positive FNAC results that would require a different study design. Specifically, nodules with benign FNAC as well as benign histology results considered to be true-negative FNAC results, and those nodules with indeterminate or malignant FNA results, but benign final histology, classified as false-positive results were not available in the study cohort. Finally, we applied a 10 mm size cut-off to determine utilization of FNAC in cancer lesions < 1 cm and its sensitivity in this subset.

### Statistical analysis

Continuous variables are reported as median with range or mean with standard deviation (SD), as indicated by the data distribution. All statistical analyses (frequencies, descriptive statistics, χ^2^) were carried out with the SPSS v25.0 software package (IBM SPSS Statistics, Armonk, NY, USA). All p-values were predetermined to be two-sided, with the level of significance set at *p* < 0.05.

## Results

**Figure 1:**
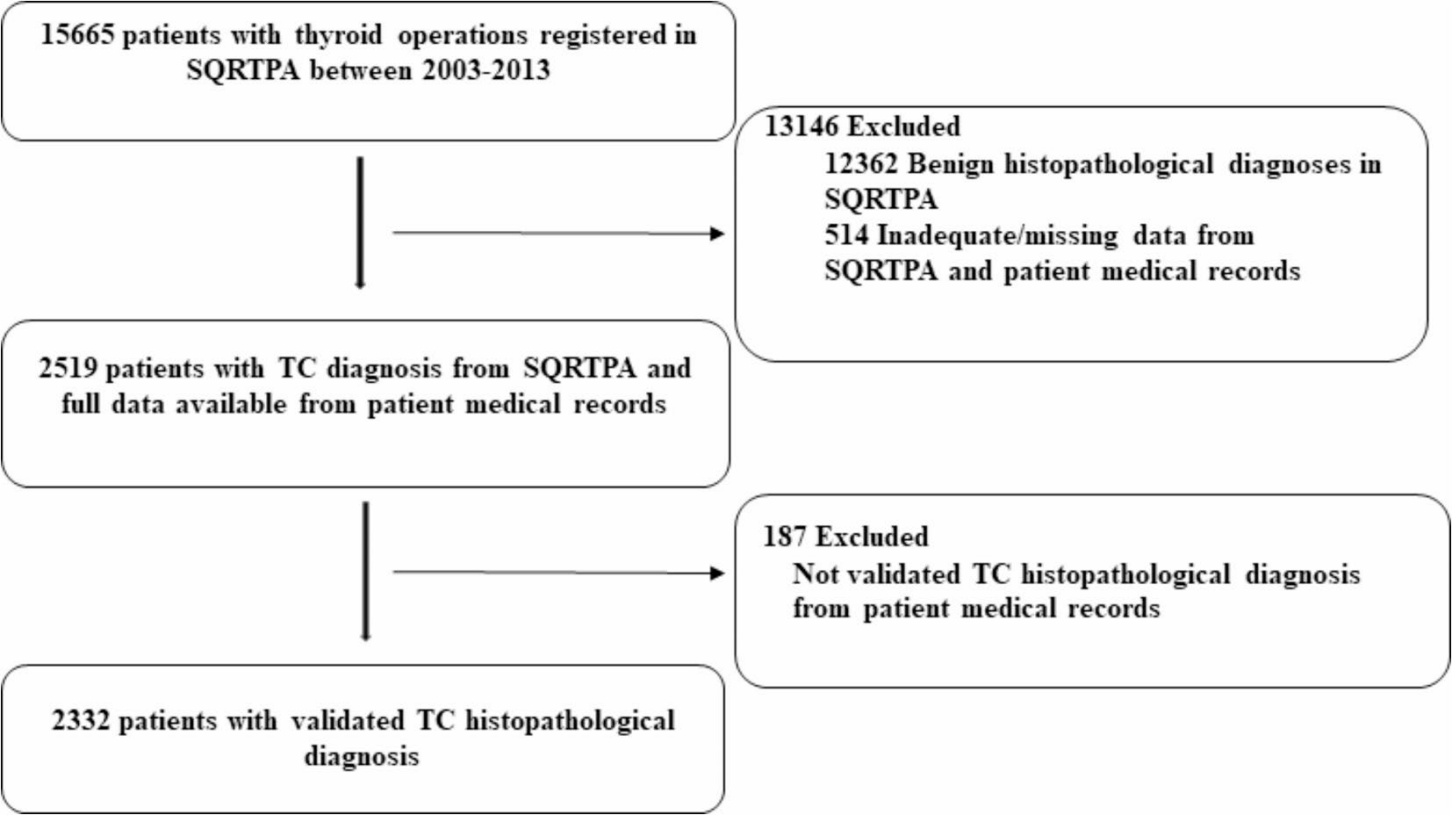
Study flow diagram. Abbreviations: TC, thyroid cancer; SQRTPA, Scandinavian Quality Register for Thyroid, Parathyroid and Adrenal surgery.

The study flow is given in Fig. 1. Among the 2519 cases operated for TC in SQRTPA, we included 2332 cases (92.6%) with validated TC histopathological diagnosis from the primary operation. Among these, 1679 patients (72%) were female and the median age at TC diagnosis was 52.3 years (range 18-94.6).

**Figure 2:**
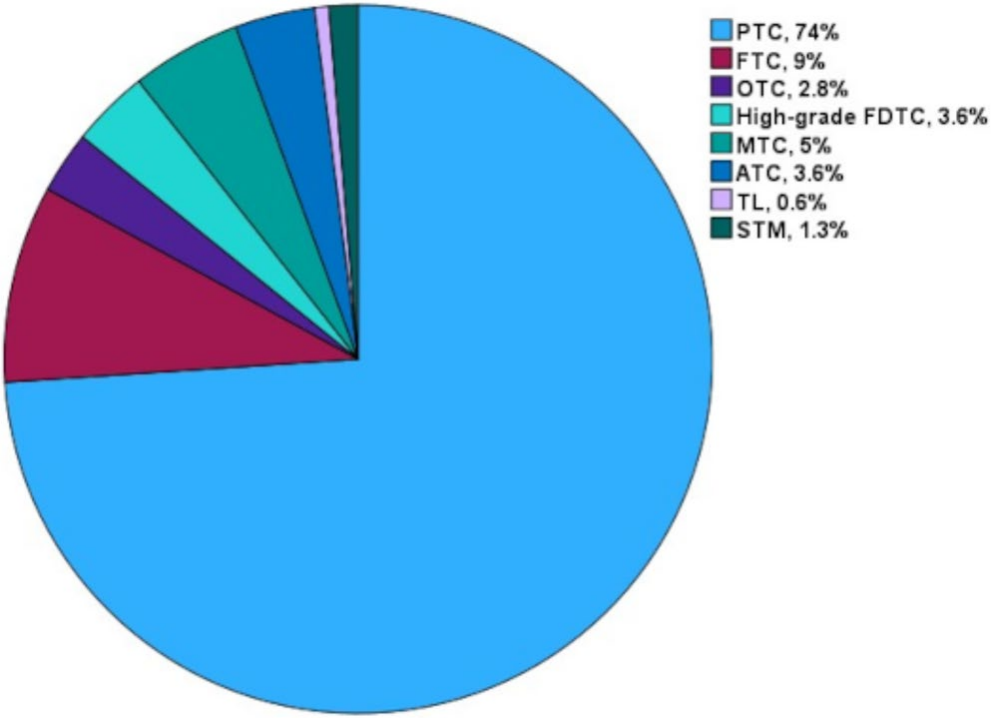
Distribution of different thyroid cancer types in Sweden. Abbreviations: TC, thyroid cancer; PTC, papillary thyroid cancer follicular; FTC, follicular thyroid cancer; OTC, oncocytic thyroid cancer; FDTC, follicular-cell derived thyroid cancer; MTC, medullary thyroid cancer; ATC, anaplastic thyroid cancer; TL, thyroid lymphoma; STM, secondary thyroid malignancies.

The distribution of TC diagnoses in relation to preoperative FNAC is demonstrated in Table 1. In addition, in Fig. 2a pie-chart of TC types’ distribution in our nation-wide cohort of TC patients during a 10-year period is given. Most cases had follicular cell–derived carcinomas, including mainly PTC (*n* = 1724; 74%), followed by FTC (*n* = 210; 9%), OTC (*n* = 66, 2.8%) and high-grade FDTC (*n* = 83; 3.6%). We encountered 117 MTC cases (5%) and 85 ATC cases (3.6%). Thyroid lymphoma was encountered in 15 cases (0.6%) and finally secondary thyroid malignancies in 32 cases (1.4%). Secondary malignancies included a broad spectrum of metastases from diverse primary tumors, such as renal cell carcinoma (*n* = 12), malignant melanoma (*n* = 1), lung cancer (*n* = 1), neuroendocrine tumor (*n* = 2), sarcoma (*n* = 4), squamous cell carcinoma (*n* = 9), mesothelioma (*n* = 1), and unknown primaries (*n* = 2).

**Table 1:** Preoperative Fine-needle aspiration cytology (FNAC) findings for each histological thyroid cancer category in the Scandinavian Quality Register for thyroid, parathyroid and adrenal surgery between 2004 and 2013.

Cytology findings	FNAC not performed	Inconclusive FNAC	Benign FNAC	AUS, FLUS or follicular neoplasm	FNAC suspicious for malignancy	Malignant FNAC
Final Histology						
PTC (*n* = 1724)	279 (16.2%)	32 (1.9%)	293 (17%)	200 (11.6%)	160 (9.2%)	749 (43.4%)
FTC (*n* = 210)	21 (10%)	3 (1.4%)	41 (19.5%)	97 (46.2%)	18 (8.6%)	29 (13.8%)
OTC (*n* = 66)	10 (15.2%)	0	6 (9.1%)	33 (50%)	8 (12.1%)	9 (13.6%)
High-grade FDTC (*n* = 52)	4 (7.7)	1 (1.9)	3 (5.8)	26 (50)	3 (5.8%)	15 (28.8)
MTC (*n* = 117)	24 (20.5%)	1 (0.9%)	6 (5.1%)	7 (6%)	6 (5.1%)	73 (62.4%)
ATC (*n* = 85)	8 (9.4%)	3 (3.5%)	3 (3.5%)	1 (1.2%)	7 (8.2%)	63 (74.1%)
Thyroid Lymphoma (*n* = 15)	4 (26.7%)	0	1 (6.7%)	1 (6.7%)	4 (26.7%)	5 (33.3%)
Metastases from other primary malignancies (*n* = 32)	2 (6.3%)	1 (3.1%)	3 (9.4%)	1 (,11%)	5 (15.6%)	20 (62.5%)

*FNAC* fine-needle aspiration cytology, *AUS* atypia of undetermined significance, *FLUS* follicular lesion of undetermined significance, *TC* thyroid cancer, *PTC* papillary thyroid cancer, *FTC* follicular thyroid cancer, *OTC* Oncocytic thyroid cancer, *FDTC* Follicular-cell derived thyroid cancer, *MTC* medullary thyroid cancer, *ATC* anaplastic thyroid cancer.

Among the 2332 TC cases of the registry, 356 (15.3%) had benign FNAC; in 41 cases (1.8%) FNAC was non-diagnostic because the smears were not satisfactory for diagnosis, whereas in 367 cases (15.7%) FNAC was not performed at all. FNAC suggestive of atypia of unknown significance (AUS), follicular lesion of unknown significance (FLUS) and/or follicular neoplasm was present in 372 cases (16%). Lesions suspicious for malignancy and malignant lesions were present in 216 (9.3%) and 980 (42%), respectively. The FNAC findings in respect of each cancer type are demonstrated in Table 1.

With regards to TNM classification, 124 patients had missing data on tumor size, 571 had T1a, 401 T1b, 540 T2, and 696 T3 and T4 tumors. Although we had some available data on extrathyroidal extension (ETE), we lacked detailed data on the extend of tumor infiltration (minimal vs. extensive ETE) in our dataset, as well as detailed data on N status (nodal dissection findings).

In TC cases subjected to FNAC, the sensitivity of FNAC was as high as 81.6%. In the subset of lesions > 1 cm the FNAC sensitivity was 86.5%, whereas in sub-centimeter lesions FNAC yielded a sensitivity as low as 61.5%. Importantly, among patients with lesions < 1 cm (*n* = 571), 186 were not subjected to preoperative FNAC at all (32.6%). In addition, a substantial number of benign FNACs (39.9%) in the whole cohort are encountered in patients with papillary thyroid microcarcinoma (PTMC). Importantly, 193 patients with cancers < 1 cm (33.2%) were subjected to nodal dissections.

On the other hand, in larger lesions such as T3 and T4 lesions subjected to FNAC, i.e. tumors ≥ 4 cm (*n* = 539) the FNAC sensitivity was 87.4%, which is comparable to this of lesions > 1 cm. Finally in the more difficult to diagnose preoperatively, subset of patients with FTC (*n* = 210), FNAC suggested FLUS or follicular neoplasm in 97 (46.2%), suspicious malignancy in 18 (8.6%) and malignancy in 29 (13.8%) cases with an overall sensitivity rate of 77.4%. For FTC lesions > 1 cm (*n* = 189), the corresponding sensitivity figure was 77.6%.

The intermediate FNAC categories AUS / FLUS, and suspicion for follicular neoplasm lesions were more often encountered in FTC (97/210) and OTC (*n* = 33/66) than PTC cases (*n* = 200 / 1724 ; *p* < 0.001), whereas FNACs suspicion for malignancy and/or malignant FNCA categories were overrepresented in PTC (*n* = 909/1724) as compared to FTC (*n* = 47/210) and OTC categories (*n* = 17/66; *p* < 0.001).

Approximately 85% of FNACs (*n* = 1981/2332) were performed using ultra-sonographic (US) guidance. In TC lesions > 1 cm, the diagnostic sensitivity of US-guided FNAC (*n* = 1504) was 86.9% as compared to 76.9% in clinically applied FNAC without US utilization (*n* = 118). Interestingly, the mean tumoral size was larger in patients subjected to US-guided FNAC (mean size = 27.9 mm, SD = 21.3) vs. those subjected to palpation-guided FNAC (mean size = 13.5, SD = 18.8, *p* < 0.001).

## Discussion

This study explored 2332 TC cases across a 10-year period from the SQRTPA registry with nation-wide validation and FNAC and histology correlation. In addition, we assessed the sensitivity of FNAC in TC cases. With regards to tumoral size, a significant proportion of tumors were < 1 cm in size (*n* = 571, 24.5%). FNAC utilization in lesions < 1 cm was limited, as 33.5% of cases was not subjected to FNAC and a proportion as high as 24.9% had benign FNACs. Importantly, PTMC is probably underreported in the national registry, as they often lack clinical significance.

In our large nation-wide series of TC patients with preoperative FNAC results meticulously linked with those of surgical histology, we show conclusively that FNAC is performed in most patients (85%) operated for TC in Sweden and that FNAC as a diagnostic modality yielded a sensitivity of approximately 82% in the whole cohort and as high as 87% for TC lesions > 1 cm. Furthermore, we describe an overall relatively higher false-negative rate in lesions < 1.0 cm with available FNAC results as compared to larger lesions, possibly due to difficulty aspirating smaller lesions with a resultant sampling of adjacent normal tissue^14^.

Previous studies have attempted to link tumor size to FNAC accuracy, specifically false-negative rates, with conflicting results^14,15^. Nevertheless, using sonographic criteria in nodules > 1 cm, several studies have shown that larger size is not a significant predictor of malignancy^16,17^. Shrestha et al. have described a tendency toward a higher false-negative rate in lesions < 1cm^18^. Accordingly, in our study, the diagnostic sensitivity of FNAC for lesions < 1 cm was as high as 62%, implying that clinicians should be aware of potential sampling error in sub-centimeter lesions. However, due to the inherent limitations of our study in respect of its design with the inclusion of TC cases only and the lack of benign histologies, we were not able to further assess diagnostic accuracy and specifity of FNAC in thyroid nodules diagnostic work-up.

In patients harboring FTC/OTC > 1 cm, FNAC is suggestive of AUS/FLUS, suspicious for FN, suspicious for malignancy or malignancy with a sensitivity rate of 81.4%. Ιn particular, FNAC suggested FLUS or suspicious for FN in 48.5%, suspicious malignancy in 9.1% and malignancy in 15.2%.Though dependent on malignancy rates, lesions with intermediate cytological results i.e. AUS/FLUS or suspicious for FN with low-suspicion US features may benefit from clinical observation, whereas nodules with high-suspicion US features may require molecular testing and/or surgery^19^. However, we did not have a standardized US classification system for the TC cases included herein during the inclusion period and molecular testing has only been available recently. In addition, our data were primarily collected from SQRTPA and subsequently validated through scrutinizing FNAC and histology reports across 37 hospitals in Sweden. Clinical factors contributing to the application of preoperative palpation vs. US-guided FNAC include the lack of availability of US-guided FNAC in some Swedish hospitals during the inclusion period, and other parameters as well, such as nodule size, thyroid volume, thyroid function and finally physicians’ preference.

In our study, we were not able to assess measurements on dedicated preoperative US, however in the majority of the patients included herein, documented measurements (maximal tumor diameter) from the histopathology report were available. Approximately 85% of FNACs have been performed using US guidance, leading to a better diagnostic sensitivity of US-guided FNAC (87%) as compared to clinically applied FNAC without US utilization (sensitivity rate of 77%) in the present cohort. Accordingly, a recent systematic review and meta-analysis confirmed higher diagnostic accuracy of US-guided versus palpation FNAC for the diagnosis of malignancy in thyroid nodules^20^. Interestingly in our dataset, tumoral size was larger in patients subjected to US-guided FNAC vs. those subjected to palpation-guided FNAC, implying that nodule size itself was not the main factor that led to the application of US at FNAC sampling.

With regards to the distribution of TC diagnoses in Sweden, the majority of cases was well-differentiated TC (WDTC), including mainly PTC (74%), followed by FTC/OTC (11.8%). We encountered high grade FDTC in 3.6% of the cases. MTC was present in 5% of TC cases, ATC in 3.6%, thyroid lymphoma in 0.6% and finally secondary thyroid malignancies in 1.3%. The corresponding distribution figures from the latest SEER database report were 83.6% for PTC, 10.8% FTC, 2.2% MTC, 1.3% ATC and, 2.1% for other thyroid malignancies^21^, with evident small albeit distinct discrepancies in the frequency of the dominant PTC type and the more rare but clinically aggressive MTC and ATC cases.

After 2 decades of rapid increase between 1992 and 2009, the incidence of TC in the United States has reached a plateau and possibly started to decline until 2014^1,21^. Interestingly, increasing trends in the incidence of sub-centimeter thyroid cancers, most prone to increasing detection, began to reverse direction between 2013 and 2016^21^. These changes have occurred during a time of evolving understanding of overdiagnosis and the indolent nature of many small thyroid cancers, reflected in changing clinical practice guidelines, including recommendations against screening for thyroid cancer as well as against routine biopsy for many smaller, lower-risk nodules^22,23^. However, the inclusion period of our study precedes these changes and does not reflect the introduction of a more conservative approach in the Swedish clinical practice, as 385 out of 571 subcentimenter thyroid cancer cases (67.4%) were subjected to FNAC possibly leading to overdiagnosis and overtreatment of subclinical cases. However, 33% of cancer lesions < 1 cm in this dataset were subjected to nodal dissections, implying that they could be of clinical relevance.

In addition, a common problem in all studies examining the FNAC validity is that at least in a group of patients with benign cytology, TC may be a sudden finding in another nodule not submitted to FNAC. In our study, we scrutinized cytology and final histology reports, also with the aims to assess whether FNAC was obtained from the same nodule described in the histology report. However, as data from US reports were not scrutinized in relation to the study findings, this could be a source of confounding particularly in the subset of patients with < 1 cm TC lesions. Therefore, we have undertaken sub-analyses for lesions > 1 cm, where FNAC seems to yield higher sensitivity rates and these lesions are considered more clinically relevant. In addition, in TC lesions < 1 cm the indication for FNAC and subsequent surgery in cases of benign cytology could be multifaceted including ultrasonographic suspicion of TC, elevated calcitonin levels in cases of MTC, as well as surgery for other thyroid pathology, such as multinodular goiter and thyreotoxicosis.

Our study has some limitations. Estimates of FNAC sensitivity in TC lesions are subject to several biases, as not all patients with available FNAC undergo surgical resection and confirmatory histologic analysis, e.g. those with inconclusive, benign or intermediate FNAC categories. In our study, we only focused on TC cases and included patients undergoing surgery from SQRTPA with available histological correlation to FNAC findings after meticulous data scrutinization and nation-wide validation. Furthermore, we could not assess the specificity of FNAC in TC setting, as we only included TC cases and could not validate all true negative cases, i.e. Bethesda II categories in benign histopathology reports from SQRTPA (*n* = 12362). Clinical factors contributing to the decision to refer for surgery with a high clinical suspicion for TC despite the absence of FNAC or in cases of an inconclusive or benign FNAC may have indeed confounded our results. Such factors include suspicious features on US, nodule growth, local compressive symptoms, nodule size, and patient concern or preference. Nevertheless, our dataset also includes non-FNAC considerations leading to surgical referral and final malignant histology.

We chose to classify indeterminate FNAC results as true positive for the included TC cases at the time of surgery. This practice may indeed contribute to higher figures of FNAC sensitivity. However, we felt it more accurate to label a sufficient FNAC resulting in a decision to refer to surgery as positive, as currently in the Swedish clinical practice intermediate FNACs most often lead to diagnostic procedures, commonly hemi-thyroidectomies. As the period of inclusion of TC patients was between 2004 and 2013, the cytological criteria for the evaluation of thyroid lesions, including those of WDTC, have evolved over the last decade possibly accounting for confounding in terms of cytological misclassification when recoding cytological reports obtained prior to the Bethesda adoption in Sweden. Therefore, the lack of central cytology re-assessment that concerns both inter-study concordance for FNAC assessment in different hospitals and evolution of cytological criteria over time constitute a major limitation of our study. However, most TC cases in the present cohort were included from high volume Swedish endocrine surgery centers with cytologists/pathologists, specialized in endocrine disorders.

## Conclusions

FNAC is performed in most patients (85%) operated for TC in Sweden and retains its value as a tool in TC diagnostic work-up with a sensitivity rate of 82%. A substantial number of benign FNACs (39.9%) in this cohort were encountered in patients with lesions < 1 cm, partly owing to PMTC incidentalomas that often lack clinical significance. Overall, FNAC sensitivity for clinically relevant TC lesions > 1 cm was as high as 87%. In patients harboring the more difficult to diagnose preoperatively FTC, FNAC exhibited a sensitivity of 77%, albeit including intermediate cytology categories such as AUS, FLUS and suspicious for FN as true positive results. Finally, most FNACs (85%) were performed applying US guidance, leading to a better diagnostic sensitivity of FNAC as compared to clinically applied FNAC without US utilization.

## Electronic supplementary material

Below is the link to the electronic supplementary material.

Supplementary Material 1

Supplementary Material 2

## Data Availability

The registry data underlying this article will be shared upon reasonable request to the corresponding author.
