# Supplementary material for: Diagnostic sensitivity of fine-needle aspiration cytology in thyroid cancer
Source: Sci Rep. 2024 Oct 16;14:24216. doi: 10.1038/s41598-024-75677-7 (PMC11484879; doi:10.1038/s41598-024-75677-7)
Supplement: Supplementary file 1 — Supplementary Material 1 [file 41598_2024_75677_MOESM1_ESM.docx]

**Questionnaire**

Preoperatively undiagnosed thyroid malignancy

Preoperatively suspected thyroid malignancy

– with benign final response PAD

Preoperatively benign or not performed cytology/biopsy with malignant final response PAD

Operation ID

Patient ID

TUMOR QUESTIONS:

Histological diagnosis (control of registration)______________________________

1. Size of tumor__________ (mm)

2. Multicentricity YES NO

3. Macroscopic radicality YES NO

4. Microscopic radicality YES NO

5. Corresponds to the position of the tumor that preop.

the palpation finding YES NO

Possible comments______________________________________________________________

Preoperative Diagnostics

5. Cytology YES NO

If yes:

6. Cytological puncture via ultrasound YES NO

7. Is the cytology reviewed postoperatively YES NO

If yes:

8. Still benign cytology at follow-up YES NO

9. Biopsy YES NO

If yes:

10. Biopsy via ultrasound YES NO

11. Is the biopsy reviewed postoperatively YES NO

If yes:

12. Still benign biopsy at follow-up YES NO

QUESTIONS REGARDING OPERATION:

13. Suspected malignancy perop YES NO

14. Are frozen sections used per operation YES NO

15. Was performed later supplementary op YES NO

Ev.

Comment_____________________________________________________________________________________________________________________________________________

Patrik Lind (student), Göran Wallin

Submitted no later than October 15, 2015 to:

Chief physician Göran Wallin

Surgical Clinic

Örebro University Hospital

Södra Grev Rosengatan

70185 Örebro

Sweden
