# Supplementary material for: Diagnostic sensitivity of fine-needle aspiration cytology in thyroid cancer
Source: Sci Rep. 2024 Oct 16;14:24216. doi: 10.1038/s41598-024-75677-7 (PMC11484879; doi:10.1038/s41598-024-75677-7)
Supplement: Supplementary file 2 — Supplementary Material 2 [file 41598_2024_75677_MOESM2_ESM.doc]

# ENKÄT

**Preoperativt ej diagnosticerad tyreoidea malignitet**

**Preoperativt misstänkt tyreoidea malignitet**

**– med benignt slutsvar PAD**

**Preoperativt benign eller ej utförd cytologi/biopsi**

## Operations ID

## Patient ID

TUMÖRFRÅGOR:

Histologisk diagnos(kontroll av registrering)______________________________

1. Storlek av tumör__________ (mm)
2. Multicentricitet JA NEJ
3. Makroskopisk radikalitet JA NEJ
4. Mikroskopisk radikalitet JA NEJ
5. Motsvarar läget av tumören det preop.

palpationsfyndet JA NEJ

Ev.kommentar_________________________________________________________

Preoperativ Diagnostik

5. Cytologi JA NEJ

**Om ja:**

6.Cytologisk punktion via ultraljud JA NEJ

7. Är cytologin eftergranskad postoperativt JA NEJ

**Om ja:**

8. Fortfarande benign cytologi vid eftergranskning JA NEJ

9. Biopsi JA NEJ

**Om ja:**

10. Biopsi via ultraljud JA NEJ

11. Är biopsi eftergranskad postoperativt JA NEJ

**Om ja:**

12. Fortfarande benign biopsi vid eftergranskning JA NEJ

**FRÅGOR AVSEENDE OPERATION:**

13. Misstänktes malignitet perop JA NEJ

14. Användes fryssnitt per op JA NEJ

15. Utfördes senare kompletterande op JA NEJ

Ev.

Kommentar_____________________________________________________________________________________________________________________________________________

Lind (student), Göran Wallin

Insändes senast 15 oktober 2015 till:

Överläkare Göran Wallin

Kirurgiska Kliniken

Örebro Universitetssjukhus

Södra Grev Rosengatan

70185 Örebro

Sverige
